# Effect of a Single Session of Intermittent Hypoxia on Erythropoietin and Oxygen-Carrying Capacity

**DOI:** 10.3390/ijerph17197257

**Published:** 2020-10-04

**Authors:** Mercedes J. Nagel, Caitlin P. Jarrard, Sophie Lalande

**Affiliations:** Department of Kinesiology and Health Education, The University of Texas at Austin, Austin, TX 78751, USA; mnagel@utexas.edu (M.J.N.); cpjarrard@utexas.edu (C.P.J.)

**Keywords:** intermittent hypoxia, erythropoietin, hemoglobin mass, arterial oxygen saturation

## Abstract

Intermittent hypoxia, defined as alternating bouts of breathing hypoxic and normoxic air, has the potential to improve oxygen-carrying capacity through an erythropoietin-mediated increase in hemoglobin mass. The purpose of this study was to determine the effect of a single session of intermittent hypoxia on erythropoietin levels and hemoglobin mass in young healthy individuals. Nineteen participants were randomly assigned to an intermittent hypoxia group (Hyp, *n* = 10) or an intermittent normoxia group (Norm, *n* = 9). Intermittent hypoxia consisted of five 4-min hypoxic cycles at a targeted arterial oxygen saturation of 90% interspersed with 4-min normoxic cycles. Erythropoietin levels were measured before and two hours following completion of the protocol. Hemoglobin mass was assessed the day before and seven days after exposure to intermittent hypoxia or normoxia. As expected, the intermittent hypoxia group had a lower arterial oxygen saturation than the intermittent normoxia group during the intervention (Hyp: 89 ± 1 vs. Norm: 99 ± 1%, *p* < 0.01). Erythropoietin levels did not significantly increase following exposure to intermittent hypoxia (Hyp: 8.2 ± 4.5 to 9.0 ± 4.8, Norm: 8.9 ± 1.7 to 11.1 ± 2.1 mU·mL^−1^, *p* = 0.15). Hemoglobin mass did not change following exposure to intermittent hypoxia. This single session of intermittent hypoxia was not sufficient to elicit a significant rise in erythropoietin levels or hemoglobin mass in young healthy individuals.

## 1. Introduction

Cardiorespiratory fitness, assessed by measuring maximal oxygen consumption, represents the ability of the cardiovascular system to transport and use oxygen during maximal exercise. Oxygen transport from the lungs to the exercising muscles is achieved through the binding of oxygen to hemoglobin, an iron-containing protein in the red blood cell. Thus, hemoglobin mass represents the oxygen-carrying capacity of the blood and strongly correlates with maximal oxygen consumption [1]. Interventions that increase hemoglobin mass would therefore improve the oxygen-carrying capacity and, ultimately, maximal oxygen consumption. The development of novel interventions to improve oxygen-carrying capacity remains particularly important for populations with limited exercise tolerance that do not meet recommended physical activity levels, such as patients with type 2 diabetes [2,3]. It was previously reported that patients with type 2 diabetes have a 20% lower blood volume than healthy individuals [4]. The combined observation of normal hematocrit levels with a lower blood volume indicates a reduced hemoglobin mass in patients with type 2 diabetes. Thus, a reduced oxygen-carrying capacity likely contributes to the lower maximal oxygen consumption consistently observed in patients with type 2 diabetes [5,6].

Diminished oxygen availability stimulates the release of erythropoietin (EPO), a glycoprotein regulating red blood cell production, in order to restore oxygen supply to the tissues [7]. Indeed, a few minutes of exposure to hypoxia stabilizes hypoxia inducible factor-1α, which activates EPO gene transcription and EPO production [7]. The rise in serum EPO levels lasts several hours and results in the creation of reticulocytes that mature into red blood cells within 5–6 days [8]. Fifteen sessions of intermittent hypoxia, consisting of breathing hypoxic air for 3–5 min interspersed with 3 min of breathing normoxic air over a period of 3 weeks, increased hemoglobin mass and exercise tolerance in a group composed of healthy active individuals at risk for chronic obstructive pulmonary disease and patients with mild chronic obstructive pulmonary disease [9]. The authors suggested that a hypoxia-induced increase in EPO levels was the underlying mechanism for this hematological adaptation to repeated sessions of intermittent hypoxia. Indeed, each session of intermittent hypoxia likely triggered an increase in EPO levels, which is in accordance with previous findings that a 90-min single session of continuous hypoxia increased EPO levels and the number of immature red blood cells in untrained young healthy men [10]. A single session of intermittent hypoxia could therefore be sufficient to induce changes in oxygen-carrying capacity. Thus, the objective of the present study was to determine whether one session of intermittent hypoxia triggers an increase in EPO levels, and consequently hemoglobin mass, in young healthy individuals. It was hypothesized that a single session of intermittent hypoxia would induce significant increases in EPO levels, which would lead to an increased hemoglobin mass. These findings will contribute to the identification of the shortest intermittent hypoxia intervention necessary to trigger beneficial increases in oxygen-carrying capacity. 

## 2. Materials and Methods 

Healthy, non-smoking men and women were recruited to participate in the study. All participants provided informed written consent for participating in the study. The study was conducted in accordance with the Declaration of Helsinki, and the protocol was approved by the Institutional Review Board of the University of Texas at Austin (IRB study number: 2017-09-0015). Nineteen individuals were randomly assigned to an intermittent hypoxia group (Hyp, *n* = 10, 5 women) or a placebo intermittent normoxia group (Norm, *n* = 9, 5 women). Participants visited the laboratory on three occasions over a period of 8 days. During Visit 1 and 3, measures of hemoglobin mass, red blood cell volume, plasma volume, and total blood volume were performed. Visit 2 included measures of EPO levels before and 2 h following the completion of an intermittent hypoxia protocol or an intermittent normoxia protocol. Visit 2 was scheduled the day after Visit 1, and Visit 3 took place 7 days after Visit 2. Because menstrual blood loss has no impact on hemoglobin mass [11], visits were scheduled during any phase of the menstrual cycle. Average heart rate and blood pressure were calculated from 3 measures obtained following 5 min of supine rest on Visit 1 (Omron Healthcare, Inc., Lake Forest, IL, USA). Participants were asked to avoid alcohol and intense physical activities on the day prior to all visits. 

### 2.1. Intermittent Hypoxia

The intermittent hypoxia protocol consisted of five 4-min hypoxic cycles at a targeted arterial oxygen saturation of 90% interspersed with 4-min normoxic cycles. This protocol was based on the intermittent hypoxia protocol used by Burtscher et al. [9], which consisted of progressive changes in the number of hypoxic cycles (3, 4, and 5 cycles), hypoxic duration (3, 4, and 5 min), and hypoxic severity (fraction of inspired oxygen of 0.15, 0,13, and 0.12) over 3 weeks. The 4-min normoxic duration is in accordance with the half-life of 5–8 min of hypoxia inducible factor-1*α* [12], and was based on previous intermittent hypoxia protocols that used normoxic bouts lasting between 3 to 5 min [9,13]. Due to the high individual variability in hypoxic ventilatory responses, a fixed fraction of inspired oxygen can result in a wide range of arterial oxygen saturation across individuals. Moreover, arterial oxygen saturation was observed to increase over the course of a session of intermittent hypoxia of 5 cycles at a fixed oxygen level [14]. Therefore, intermittent hypoxia was not performed at a fixed oxygen level but at a targeted arterial oxygen saturation in order to induce the same level of hypoxemia for each cycle and in all participants. An arterial oxygen saturation of 90% was chosen based on our previous observations that this arterial oxygen saturation corresponds to a fraction of inspired oxygen of 0.12 ± 0.01 in young healthy individuals. This intermittent hypoxia protocol is therefore considered mild as it represents less than 10 cycles of hypoxia lasting between 15 s to 4 min at a fraction of inspired of oxygen between 0.10 to 0.14 [15].

Participants inhaled hypoxic air through a mask connected to a two-way non-rebreathing valve (Hans Rudolph, Inc, Shawnee, KS, USA), which was itself connected to a five-liter non-diffusing gas bag (Hans Rudolph, Inc, Shawnee, KS, USA). The rebreathing bag was connected to a gas tank of compressed air. Air was made hypoxic by introducing nitrogen in the breathing circuit. The flow of nitrogen was controlled to achieve an arterial oxygen saturation of 90%, as measured by pulse oximetry. Participants in the intermittent normoxia group performed the same protocol, but nitrogen was not introduced in the breathing circuit. Pulmonary gas exchange, hemodynamics, and arterial oxygen saturation were continuously measured during the intermittent hypoxia protocol and intermittent normoxia protocol.

### 2.2. Hematological Variables

Hemoglobin mass was determined using a modified version of the optimized carbon monoxide rebreathing technique [16,17]. Briefly, a venous blood draw was first obtained to measure baseline levels for carboxyhemoglobin, hematocrit, and hemoglobin (ABL 80 FLEX OSM, Radiometer, Copenhagen, Denmark). Participants then rebreathed an individually calculated volume of carbon monoxide that was introduced into a low-volume closed-circuit rebreathing system containing ambient air for a period of 2 min. A second venous blood draw was obtained 10 min following the start of the carbon monoxide rebreathing to assess the plateau in carboxyhemoglobin levels resulting from the complete mixing of carbon monoxide into the circulation [17]. Hemoglobin mass, red blood cell volume, plasma volume, and total blood volume were calculated from the change in carboxyhemoglobin levels induced by carbon monoxide rebreathing [18]. The coefficient of variation for hemoglobin mass, based on duplicate measures performed on consecutive days in five individuals, is 2.6% in our laboratory. 

### 2.3. Hemodynamics

An arterial waveform obtained by finger plethysmography from the middle finger of the left hand was continuously recorded throughout the intermittent hypoxia protocol and intermittent normoxia protocol (NOVA, Finapres Medical Systems, Amsterdam, the Netherlands). Brachial arterial blood pressure, heart rate, stroke volume, cardiac output, and total peripheral resistance were derived from the arterial waveform, a method that has been validated against invasive measures [19]. Arterial oxygen saturation was also continuously monitored by pulse oximetry (NOVA, Finapres Medical Systems, Amsterdam, Netherlands). All data were recorded in LabChart for later analysis (Powerlab, ADInstruments Inc., Colorado Springs, CO, USA). 

### 2.4. Pulmonary Gas Exchange

Breath-by-breath measures of pulmonary gas exchange were collected and analyzed every 10 s throughout the intermittent hypoxia protocol and intermittent normoxia protocol using a metabolic cart calibrated with room air and standardized gas (Ultima Cardio2, MGC Diagnostics, St. Paul, MN, USA). The pneumotachometer was mounted between the mask and the non-rebreathing valve of the breathing circuit.

### 2.5. Erythropoietin Levels

Upon arrival to the laboratory on Visit 2, a venous blood draw was obtained for later analysis of EPO levels. Previous studies consistently reported EPO levels peaking 2–3 h following termination of hypoxic bouts [10,20,21,22,23,24]; therefore, another blood draw was performed 2 h following the completion of the intermittent hypoxia protocol or intermittent normoxia protocol. Following collection, blood was centrifuged and serum aliquoted and stored at −80 °C for subsequent analyses. EPO levels were determined using an enzyme-linked immunosorbent assay (Abcam, Cambridge, UK). 

### 2.6. Data and Statistical Analyses

Average values for each pulmonary gas exchange and hemodynamics variables were calculated for the last minute of each hypoxic and normoxic cycles. A one-way repeated measured analysis of variance was used to evaluate the effect of each cycle of intermittent hypoxia or intermittent normoxia on physiological variables. An average value was then calculated for each variable for all hypoxic cycles of the intermittent hypoxia protocol and for all normoxic cycles of the intermittent normoxia protocol. A Student’s t-test was used to evaluate the effect of group (Hyp and Norm) on participants’ characteristics, pulmonary gas exchange, and hemodynamics. A two-way analysis of variance was used to evaluate the effect of group (Hyp and Norm) and time (pre- and post-intervention) on EPO levels and hematological variables. When main effects were significant, post hoc analyses were performed using Tukey’s test. Pearson’s correlation was used to determine the relation between fraction of inspired oxygen, arterial oxygen saturation, and changes in EPO levels. An a priori calculation of sample size for a repeated measures analysis of variance (within factors) was performed (G*Power, Heinrich-Heine-Universität Düsseldorf, Germany). Using baseline EPO levels and EPO levels in response to 120 min of continuous hypoxia [21], a power of 0.80 and an alpha level of 0.05, it was determined that a minimum sample size of 6 participants was needed in the Hyp group. Significance was set at *p* ≤ 0.05. All values are reported as mean ± standard deviation.

## 3. Results

Age, weight, height, hemoglobin concentration, hematocrit levels, resting blood pressure, heart rate, and physical activity levels were not different between groups (Table 1). A significant increase in fraction of inspired oxygen was observed with repeated cycles of intermittent hypoxia (first cycle: 0.118 ± 0.008; fifth cycle: 0.123 ± 0.007, *p* ˂ 0.01). There was no difference across hypoxic cycles and normoxic cycles for any other hemodynamic and pulmonary gas exchange variables. By design, arterial oxygen saturation was lower during intermittent hypoxia than intermittent normoxia (Table 2), which was equivalent to a lower fraction of inspired oxygen (Table 3). There was no difference between intermittent hypoxia and intermittent normoxia for any other hemodynamic or pulmonary gas exchange variables (Table 2 and Table 3).

**Figure 1:**
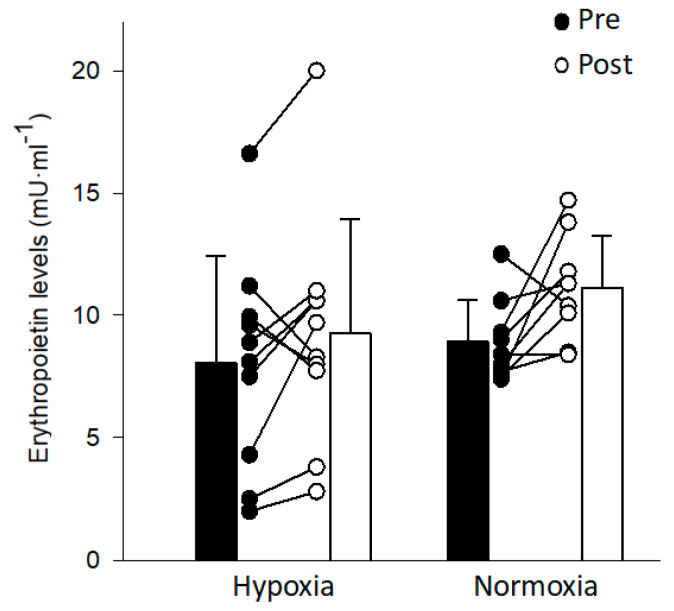
Average and individual erythropoietin levels before (pre; black bars) and 2 h following the end (post; white bars) of an intermittent hypoxia and intermittent normoxia protocol.

**Figure 2:**
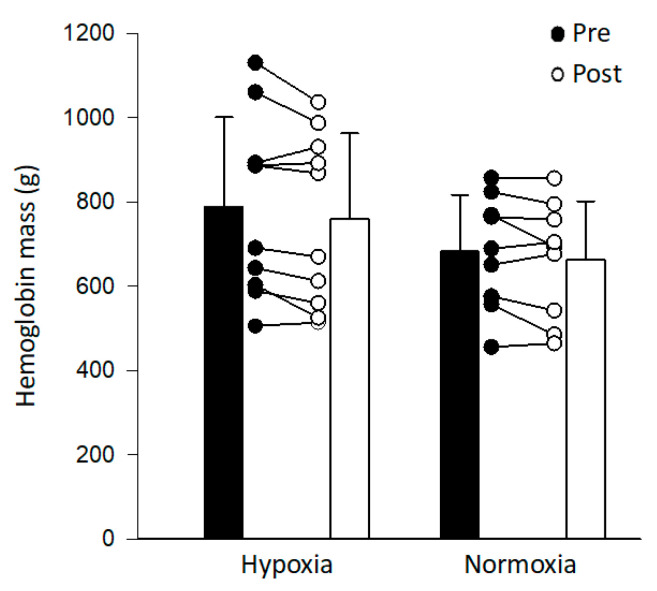
Average and individual hemoglobin mass before (pre; black bars) and 7 days following exposure to intermittent hypoxia or intermittent normoxia (post; white bars).

EPO levels did not significantly change following exposure to intermittent hypoxia (Figure 1). The coefficient of variation for the EPO assay was 11.2 ± 7.4%. There was no correlation between changes in EPO levels and arterial oxygen saturation (*r* = 0.07, *p* = 0.77) or between changes in EPO levels and fraction of inspired oxygen (*r* = 0.21, *p* = 0.39). Hemoglobin mass did not change following intermittent hypoxia or intermittent normoxia (Figure 2). Similarly, hemoglobin mass normalized to weight, red blood cell volume, plasma volume, and total blood volume did not change following intermittent hypoxia or intermittent normoxia (Table 4). Pre- and post-intervention plasma volume and total blood volume were significantly higher in the Hyp vs. Norm group (Table 4).

## 4. Discussion

The purpose of the present study was to determine whether a single session of intermittent hypoxia increases EPO levels and hemoglobin mass in young healthy individuals. Contrary to our hypothesis, one session of intermittent hypoxia, consisting of a total hypoxic duration of 20 min at an arterial oxygen saturation of 89 ± 1%, was not sufficient to induce a rise in EPO levels, resulting in an expected lack of increase in hemoglobin mass. The duration and severity of the intermittent hypoxia protocol likely contributed to the observed lack of hypoxia-induced increase in EPO levels.

Single exposures to continuous hypoxia ranging between 84 and 120 min consistently increase EPO levels [10,20,21,22,25]. Schmidt et al. [10] reported that 90 min of hypoxia increased the number of reticulocytes 2 days following the hypoxic exposure, and suggested that the higher erythrocyte production rate was caused by the increase in EPO levels. Exposure to 240 min of intermittent hypoxia, consisting of 2.5 min of hypoxia alternating with 1.5 min of normoxia for a total duration of 108 ± 6 min below an arterial oxygen saturation of 90%, elicited an increase in EPO levels similar to the rise in EPO levels observed following 120 min of continuous hypoxia [21]. Even though the total hypoxic duration was shorter for the intermittent hypoxia protocol in comparison to the continuous hypoxia protocol (108 vs. 120 min), several hypoxic episodes may have an additive effect on HIF-1*α* stabilization and, thereby, on EPO expression. The intermittent hypoxia protocol used in the present study represented a total of 20 min of hypoxic exposure and may therefore not have been sufficient to elicit increases in EPO levels. However, repeated sessions of an intermittent hypoxia protocol of similar duration, consisting of 3 to 5 hypoxic bouts lasting 3–5 min each interspersed with 3-min normoxic intervals, increased red blood cell count and hemoglobin mass in healthy and clinical populations, likely due to a hypoxia-related stimulation of erythropoiesis [9,26]. Each of these sessions likely triggered an increase in EPO levels, which translated into a significant increase in hemoglobin mass following several sessions. Based on these previous findings, it was hypothesized that the duration of the intermittent hypoxia protocol used in the present study would be sufficient to induce an increase in EPO levels. 

The effect of intermittent hypoxia on hemoglobin mass varies across populations. In endurance-trained athletes, an intermittent hypoxia protocol consisting of seven cycles of 5-min hypoxic bouts interspersed with 5-min normoxic bouts performed five times/week for four weeks failed to change hemoglobin concentration [13]. However, measures of hemoglobin concentration depend on plasma volume and are, therefore, greatly influenced by hydration status [27], which could have masked an increase in hemoglobin mass. Moreover, endurance-trained athletes have a 40–50% greater hemoglobin mass than sedentary individuals [1], which may limit further erythropoiesis in this population. On the other hand, repeated sessions of intermittent hypoxia increased hemoglobin mass and red blood cell count in clinical populations as well as in active individuals at risk for chronic obstructive pulmonary disease [9,26], suggesting that intermittent hypoxia can trigger an increase in hemoglobin mass in healthy individuals. Accordingly, a single session of 90-min of continuous hypoxia increased reticulocytes in untrained young healthy men [10]. While intermittent hypoxia has, therefore, the potential to improve oxygen-carrying capacity in patients with type 2 diabetes, the reduced hemoglobin mass of this population may be due to low levels of erythropoietin, functional erythropoietin deficiency, and/or erythropoietin resistance in this population [4]. Thus, the objective of the present study was to first determine whether a single session of intermittent hypoxia triggers an increase in EPO that leads to an increased hemoglobin mass in young healthy individuals. 

Few studies investigated the effect of hypoxic severity on EPO levels. Exposure to simulated altitudes of 3000 and 4000 m significantly increased EPO levels in six young men [25]. Despite observing a tendency for greater increase in EPO levels with increasing altitude, there was no significant effect of hypoxic severity on EPO levels. Nonetheless, the authors described an inverted relation between alveolar partial pressure of oxygen and the relative change in the production rate of EPO. Similarly, exposure to 4200 and 4800 m, representing a fraction of inspired oxygen of approximately 0.125 and 0.115, significantly increased EPO levels in eight young men [23]. While the average increase in EPO levels tended to be greater with increasing hypoxic severity (52% at 4800 vs. 43% at 4200 m), there was also no significant effect of hypoxic severity on EPO levels due to the large individual variability in response to these fixed oxygen levels. The intermittent hypoxia protocol used in the present study induced a similar fraction of inspired oxygen of 0.120 ± 0.008 in young healthy individuals. Thus, the severity of our intermittent hypoxia protocol was sufficient to elicit an increase in EPO levels. While the fraction of inspired oxygen used in the present study was also similar to the fraction of inspired oxygen (between 0.14 and 0.10) used during repeated sessions of intermittent hypoxia that increased red blood cell count [26], it did not elicit the same level of hypoxemia in our young healthy individuals (89 ± 1%) compared to older individuals and patients with coronary artery disease (approximately 80%). Therefore, a lower targeted arterial oxygen saturation, and not a similar fraction of inspired oxygen, may be needed to trigger a release of EPO in young healthy individuals.

A potential limitation of the present study is the collection of only one data point to determine EPO levels following exposure to intermittent hypoxia. A time lag of 2–6 h exists before de novo EPO appears in serum [28]. Previous studies consistently reported EPO levels peaking 2–3 h following termination of continuous hypoxic bouts [10,20,21,22,23,24]; therefore, EPO measurements were performed 2 h following the end of the intermittent hypoxia protocol. However, when considering the duration of the hypoxic exposure, EPO levels consistently peaked 4–4.5 h following the onset of the continuous hypoxic bouts [10,20,21,22,23]. The intermittent hypoxia protocol used in the present study was shorter than these previous continuous hypoxic bouts, which resulted in EPO measurements being performed less than 3 h after the onset of the intermittent hypoxic exposure. It is therefore possible that blood draws were performed too early to detect the peak in EPO levels. However, while the collection of a single data point limited our ability to detect the peak in EPO levels, a rise in EPO should have been observed 3 h following the onset of the intermittent hypoxia protocol. Thus, the similar EPO levels between intermittent hypoxia and intermittent normoxia suggest that the intermittent hypoxia protocol did not trigger a release of EPO. Of note, Klausen et al. [29] reported significant diurnal variations of EPO concentration, with EPO concentration increasing by 15% from mid-morning to late afternoon. All baseline EPO measurements were performed between 8h and 12h; therefore, diurnal variations did not mask an increase in EPO levels induced by intermittent hypoxia. In fact, the average 20% increase in EPO levels observed in both groups likely reflects a normal diurnal variations of EPO concentration.

The intermittent hypoxic protocol used in the present study did not significantly affect any variables of pulmonary gas exchange or hemodynamics in young healthy individuals. The lack of significant change in ventilation contradicts previous findings that intermittent hypoxia protocols of five 6-min hypoxic cycles at oxygen levels of 10% or at a targeted arterial oxygen saturation of 80% increases ventilation [14,30], through an increase of approximately 250 mL in tidal volume [14]. While not significant, tidal volumes were approximately 150 mL greater with intermittent hypoxia in comparison to intermittent normoxia (Table 3). Most importantly, intermittent hypoxia did not affect blood pressure, which is in agreement with previous findings that repetitive bouts of normobaric, poikilocapnic hypoxia, consisting of 5–6 min at a fraction of inspired oxygen of 0.10 or at an arterial oxygen saturation of 80%, did not affect arterial blood pressure in young healthy individuals [14,30,31].

## 5. Conclusions

In conclusion, a 40-min session of intermittent hypoxia at a targeted arterial oxygen saturation of 90% was not sufficient to elicit a rise in EPO levels or hemoglobin mass in young healthy men and women. Nonetheless, these findings represent an important first step in the design of the shortest intermittent hypoxia protocol to trigger the release of EPO. Future studies using a protocol of greater hypoxic severity and longer duration, such as an arterial oxygen saturation of 80% with few additional hypoxic cycles, are needed to determine whether a single session of intermittent hypoxia can trigger an increase in EPO levels in both healthy individuals and clinical populations. Future studies are also needed to determine the minimum number of sessions necessary to induce significant increases in hemoglobin mass. Thus, the present findings provide an important foundation for the development of a novel, non-pharmacological intervention to improve oxygen-carrying capacity, and thereby cardiovascular health, in individuals with exercise limitations. 

## Figures and Tables

**Table 1:** Participants’ characteristics.

Variable	Hypoxia	Normoxia
Age (years)	24 ± 2	24 ± 6
Weight (kg)	73.6 ± 13.3	70.7 ± 11.4
Height (cm)	176 ± 11	171 ± 10
Hemoglobin (g·dl^−1^)	13.9 ± 1.6	14.3 ± 1.3
Hematocrit (%)	42.8 ± 4.7	43.7 ± 3.9
Systolic blood pressure (mmHg)	115 ± 11	120 ± 16
Diastolic blood pressure (mmHg)	69 ± 8	72 ± 9
Resting heart rate (bpm)	65 ± 8	64 ± 7
Physical activity levels (hours·week^−1^)	4.8 ± 2.4	4.4 ± 2.6

**Table 2:** Average values for hemodynamics variables for the last minute of the five cycles of intermittent hypoxia and intermittent normoxia.

Variable	Hypoxia	Normoxia
Systolic blood pressure (mmHg)	126 ± 9	129 ± 16
Diastolic blood pressure (mmHg)	73 ± 7	75 ± 11
Heart rate (bpm)	74 ± 9	68 ± 8
Mean arterial pressure (mmHg)	90 ± 6	93 ± 12
Stroke volume (mL)	84 ± 20	82 ± 15
Cardiac output (L·min^−1^)	6.2 ± 1.5	5.6 ± 1.2
Total peripheral resistance (mmHg·L^−1^·min^−1^)	15.9 ± 4.2	17.7 ± 4.5
Arterial oxygen saturation (%)	88.5 ± 1.3 *	98.5 ± 1.0

* *p* ˂ 0.05 between Hypoxia and Normoxia.

**Table 3:** Average values for variables of pulmonary gas exchange for the last minute of the five cycles of intermittent hypoxia and intermittent normoxia.

Variable	Hypoxia	Normoxia
Fraction of inspired oxygen	0.120 ± 0.008 *	0.209 ± 0.001
End-tidal CO_2_ (mmHg)	34.6 ± 2.0	36.7 ± 3.7
Ventilation (L·min^−1^)	8.5 ± 2.5	7.5 ± 1.6
Tidal volume (mL)	642 ± 247	500 ± 132
Respiratory rate (breaths·min^−1^)	14.8 ± 4.4	15.7 ± 2.0

* *p* ˂ 0.05 between Hypoxia and Normoxia.

**Table 4:** Hematological variables before and after intermittent hypoxia and intermittent normoxia.

Variable	Hypoxia		Normoxia	
	Pre	Post	Pre	Post
Hemoglobin mass (g·kg^−1^)	10.6 ± 1.4	10.2 ± 1.4	9.7 ± 1.6	9.4 ± 1.5
Red blood cell volume (mL)	2420 ± 646	2330 ± 619	2093 ± 412	2035 ± 420
Plasma volume (mL)	3730 ± 583 *	3592 ± 506 *	3151 ± 508	3044 ± 481
Total blood volume (mL)	6150 ± 1149 *	5923 ± 1057 *	5244 ± 839	5080 ± 840

* Main effect for group: *p* ˂ 0.05 between Hypoxia and Normoxia.
